# Testing the stability of internal quality controls stored in the automated Atellica Solution refrigerated storage module

**DOI:** 10.1515/almed-2023-0015

**Published:** 2023-04-11

**Authors:** Patricia Rayo Hidalga, Carlos Domingo Bautista, Rosa Fernandez Bonifacio, Jose L. Bedini Chesa, Naira Rico Santana

**Affiliations:** Área de Preanalítica, Centro Diagnóstico Biomédico, Hospital Clínic, Barcelona, Spain; Laboratorio Core, Centro Diagnóstico Biomédico, Hospital Clínic, Barcelona, Spain

**Keywords:** Atellica Solution, quality control, refrigerated storage module, stability, total change limit

## Abstract

**Objectives:**

Atellica Solution (AS) is a platform that incorporates immunoassay and chemistry modules. AS is fitted with a refrigerated storage module (RSM) for internal quality controls (QC). The objective of this study was to assess the maximum permissible storage time in AS for QCs.

**Methods:**

A total of 48 analytes were tested using QC materials: Liquid Assayed Multiqual (MQ), Liquichek Immunology (LI), Liquichek Lipids (LL), and Liquichek Urine Chemistry (UC). The percentage of variation between results (Xt%) was calculated as the difference between the mean value of the triplicate performed at every time point of the study (Xt) and the average of the triplicate performed in the baseline time (Xo). Stability was assessed based on the total change limit (TCL), which combines analytical and biological variation: TCL=±√((1.65 * CVa)^2^ + (0.5 * CVb)^2^).

**Results:**

A total of 40 of the 48 analytes tested remained stable at the end of the study. In relation to MQ and UC QCs, 32 of the 39 analytes remained stable for the whole study period (15 days) except for alkaline phosphatase, aspartate aminotransferase, calcium, lactate dehydrogenase, and total bilirubin in MQ, and chlorine and glucose in UC. In LI and LL QCs, eight of the nine analytes were stable throughout the 20 days of the study, except transferrin in LI.

**Conclusions:**

The Atellica Solution refrigerated storage module is a reliable system for the storage of quality control materials.

## Introduction

Clinical laboratories have to process a high volume of samples in an optimal time to help clinicians provide early diagnosis and monitor the course of patient disease. In addition, the quality of results should be guaranteed by the use of internal and external controls.

Atellica Solution is a platform developed by Siemens Healthineers (Tarrytown, NY) that combines immunoassay and chemistry modules. One of the advantages of this analyzer is its capacity to store inside internal quality controls (QCs) and calibrators through the refrigerated storage module (RSM) located in the sample handler module. This enables automation of QC processing. The RSM maintains temperature at 2–8 °C and has a maximum storage capacity of 60 QCs and/or calibrators. The containers used for them are 5 mL tubes that are loaded to the analyzer uncapped. The RSM has magnetic caps that cover the position where every tube is located to avoid evaporation. QCs can be stored for some days and storage time is set by the user.

The analyzer must guarantee the stability of QC materials while they are used. Loss of stability may lead to QC repetition and verification, which increases the workload, delays sample processing, and as a consequence there is a higher consumption of controls and reagents. QC supplier should establish recommendations about the maximum permissible storage time in the refrigeration module. However, it is always recommended that each laboratory performs its own stability test. ISO 15189:2012 recommends that internal quality control materials are assayed regularly, according to system stability [1]. The objective of this study was to assess, for each analyte, the maximum permissible storage time of the QC materials stored inside the AS RSM.

## Materials and methods

### Sample selection and processing

The study was conducted at the Core Laboratory in the Hospital Clínic of Barcelona in February 2019. During the study, the same AS analyzer and reagent lots were used. The number of calibrations was minimized to prioritize lot calibration over cartridge calibration, to the extent possible.

Four QC materials of each two levels were used: Liquid Assayed Multiqual (MQ) levels 2 and 3 of lot 45800; Liquichek Immunology (LI) levels 1 and 2 of lot 66370T; Liquichek Lipid (LL) levels 1 and 2 of lots 57560 and 57550, respectively; and Liquichek Urine Chemistry (UC) levels 1 and 2 of lot 66790T. All were manufactured by Bio-Rad Laboratories (Irvine, CA). During the study, the same QC lots were used. MQ and LL QC vials were transferred to 5 mL tubes, loading 3 mL of QC into each tube. In LI and UC QC, QC materials were ready for use in 3-mL and 4-mL tubes, respectively. Five tubes of each MQ QC level and three tubes of each UC, LI, and LL QC level were stored in the RSM. The number of tubes was established based on the panel of assays requested foreseeing a higher consumption in MQ QC. MQ and UC QC materials were stored in the RSM for 15 days, whereas LI and LL QC materials were stored for 20 days. Differences in storage time were due to the control material consumption as broader panels of analytes were assayed in MQ and UC. As a result, these control materials were consumed earlier than LI and LL. Each QC material was tested in triplicate once daily to avoid potential random pipetting errors and ensure the reliability of results. Before each run, QC materials were tested to ensure the comparability of results. The analytes that did not meet the quality specifications established by our laboratory (mean ± 2 * SD) were calibrated and controlled again. The zero time point was defined as the time at which QC materials were first loaded. The value obtained at this time point was established as the reference value.

To determine whether AS methods had an adequate analytical imprecision, CVa was calculated for each analyte using the QC materials tested to ensure the comparability of results over the study. Following SEQC^ML^ Analytical Quality Assurance Workgroup recommendations, we used quality specifications based on biological variation [2].

### Stability testing

A total of 48 biochemical analytes were tested. MQ control included uric acid (UA), alanine aminotransferase (ALT), albumin (ALB), amylase (Amylas), aspartate aminotransferase (AST), direct bilirubin (DBil), total bilirubin (TBil), calcium (Ca), chlorine (CL), total cholesterol (TChol), HDL-cholesterol (HDL-C), LDL-cholesterol (LDL-C), creatinine (Crea), creatine kinase (CK), alkaline phosphatase (ALP), phosphate (IP), glucose (Glu), gamma-glutamyltransferase (GGT), iron (Iron), lactate dehydrogenase (LDH), magnesium (Mg), potassium (K), total protein (TP), sodium (Na), triglycerides (Trig), and urea nitrogen (UN). In LI control: haptoglobin (Hapt), transferrin (Trf), C-reactive protein (CRP), prealbumin (preALB), complement C3 (C3), and complement C4 (C4). In LL control: apolipoprotein A1 (APO A1), apolipoprotein B (APO B), and high-sensitivity C-reactive protein (hsCRP). Finally, the UC control included microalbumin (microALB), Amylas; Ca, CL, Crea, Glu, IP, K, Mg, Na, UA, UN, and TP.

Following each analytical run, QC aliquots were automatically capped and stored in the AS RSM.

### Data analysis

The percentage of variation between results (Xt%) was calculated as the difference between the mean of the triplicate performed at every time point of the study (Xt) and the mean of the triplicate performed in the baseline time (Xo). Difference was expressed as a percentage difference: Xt%=(Xt/Xo) * 100 [3]. Stability was assessed based on the Total Change Limit (TCL), which combines analytical and biological variation: TCL=±√((1.65 * CVa)^2^ + (0.5 * CVb)^2^). The TCL criterion has been used in previous stability studies conducted by our working group [3, 4]. CVa is the coefficient of analytical variation for each analyte and it was calculated from internal QC data obtained in the previous 5 months from the date of the study. CVb is defined as within-subject biological variation for each analyte obtained from the *European Federation of Clinical Chemistry and Laboratory Medicine* (EFLM) database [5]. In the case of analytes not listed by the EFLM, CVbs were obtained from desirable specifications from Dr. Carmen Ricos’ biological variation database [6] (Table 1). For CL and Gluc in UC control, since no database provides CVb for these magnitudes in urine, only the analytical variation (CVa) criterion was used for TCL calculation. In these cases, the following formula was used: TCL=±√(1.65 * CVa)^2^.

**Table 1:** Analytical and biological variation coefficient of the analytes included in the study.

Analyte	CVa% (s) 5 months	CVa% (o) 5 months	CVb% (s)	CVb% (o)
ALB	1.9	8.4	2.6	
ALT	1.5		10.1	
ALP	2.3		5.3	
Amylas	1.1	2.1	6.6	94^a^
APO A1	2.9		5.4	
APO B	4.2		7.4	
AST	2.3		9.6	
C3	2.9		4.6	
C4	1.7		6.9	
Ca	2.3	3	2.1^a^	27.5^a^
TChol	1.3		5.3	
CK	1.8		15	
CL	1.3	1.4	1.1	-
Crea	2.2	1.5	4.5	24
cHDL	3.1		5.8	
DBil	2.8		36.8^a^	
cLDL	1.6		8.3	
GGT	2.7		9.1	
Glu	0.9	1.6	5	-
Hapt	2.3		8.6	
hsCRP	11.9		58.9	
IP	1.6	2.1	7.8	18^a^
Iron	1		26.5^a^	
K	0.7	1.8	4.1	24.4^a^
LDH	2.2		5.2	
Mg	3.3	5	2.9	38.3^a^
microALB		7.9		36
Na	0.5	1.8	0.5	28.7^a^
Tbil	1.7		21.8^a^	
CRP	3.7		34.1	
preALB	5.5		10.9^a^	36^a^
TP	2.1	2	2.6	35.5^a^
Trig	1.6		20	
Trf	2.8		3.9	
UA	0.8	2.3	8.6^a^	16.8^a^
UN	1.7	3.4	13.9	17.4^a^

CVa% (s), coefficient of analytical variation in serum; CVa% (o), coefficient of analytical variation in urine; CVb% (s), within-subject biological variation in serum; CVb% (o), within-subject biological variation in urine; (^a^): values obtained from the biological variation database of Dr C. Ricos; ALB, albumin; ALT, alanine aminotransferase; ALP, alkaline phosphatase; Amylas, amylase; APO A1, apolipoprotein A1; APO B, apolipoprotein B; AST, aspartate aminotransferase; C3, complement C3; C4, complement C4; Ca, calcium; TChol, total cholesterol; CK, creatine kinase; CL, chlorine; Crea, creatinine; HDL-C, HDL-cholesterol; DBil, direct bilirubin; cLDL, cholesterol-LDL; GGT, gamma-glutamyltransferase; Glu, glucose; Hapt, haptoglobin; hsCRP, high sensitivity C-reactive protein; IP, phosphate; K, potassium; LDH, lactate dehydrogenase; mg, magnesium; microALB, microalbumin; Na, sodium; TBil, total bilirubin; CRP, C-reactive protein; preALB, prealbumin; TP, total protein; Trig, triglycerides; Trf, transferrin; UA, uric acid; UN, urea nitrogen.

Loss of stability was confirmed when Xt% exceeded the TCL. Maximum permissible storage time was defined for each magnitude as the time point from which Xt%, for one or both control levels, exceeded TCL during more than two consecutive days. From that time point, QC was not stable for that specific analyte.

## Results

Analytical imprecision (CVa) was acceptable for the QC materials tested for 15 (in the case of MQ and UC) and 20 days (in the case of LI and LL) and in the previous 5 months. The acceptance criterion relied on compliance with quality specifications based on biological variation (BV). All analytes met optimal quality specifications, except for ALP, Crea, LDH, and preAlb, which met desirable specifications; ALB, APO A1, APO B, C3, cHDL, and Trf, which complied with minimal specifications; and Ca, CL, Mg, and Na, which did not meet specifications based on biological variation but had a CVa below that of their peer group. Peer group CVa was extracted from the 2019 FPCQLC External Quality Program.

**Figure 1:**
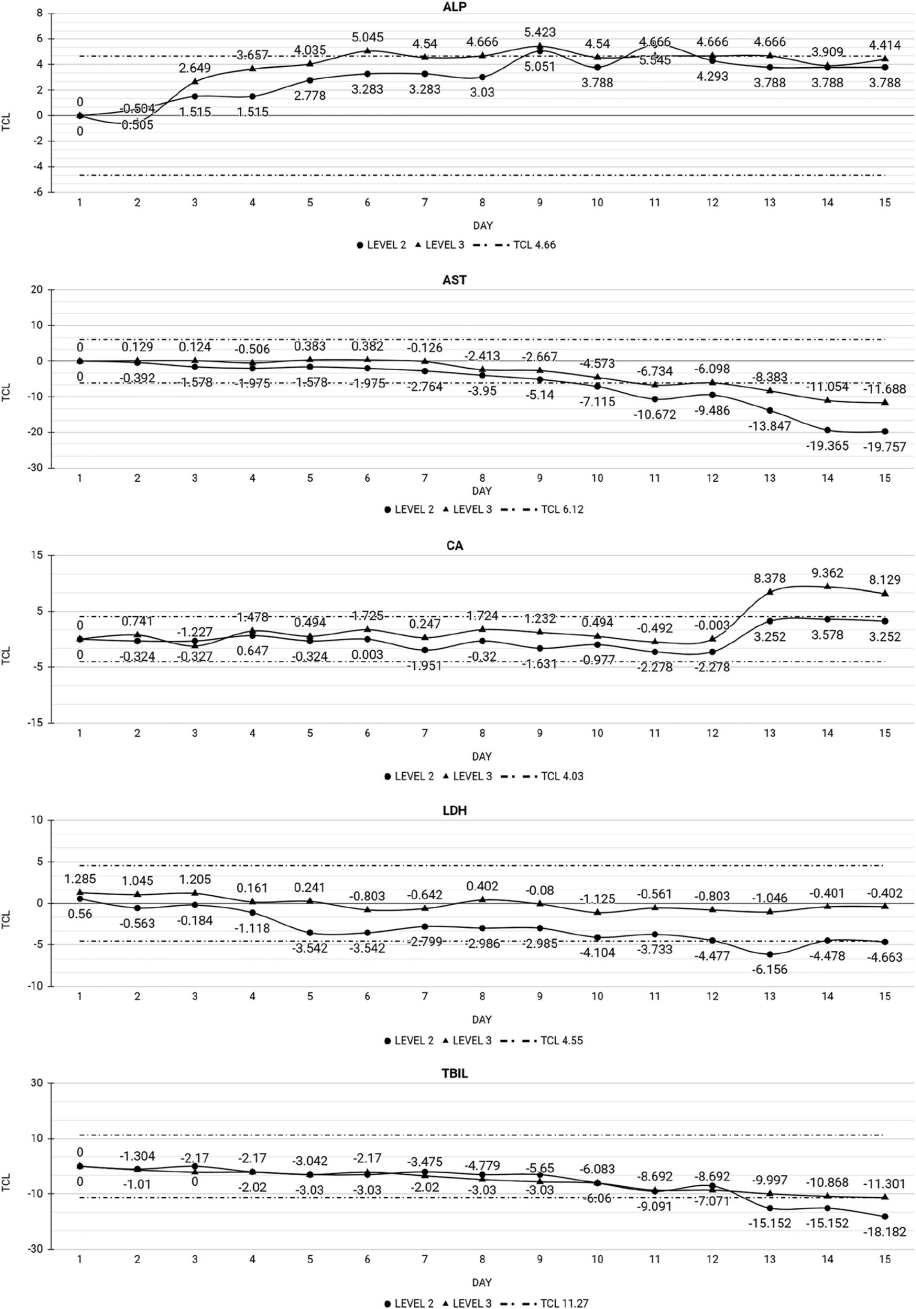
Analytes that lost stability in the MQ QC throughout the 15 days of study. ALP, alkaline phosphatase; AST, aspartate aminotransferase; CA, calcium; LDH, lactate dehydrogenase; TBIL, total bilirubin; TCL, total change limit; level 2, medium-level MQ QC; level 3, high-level MQ QC.

**Figure 2:**
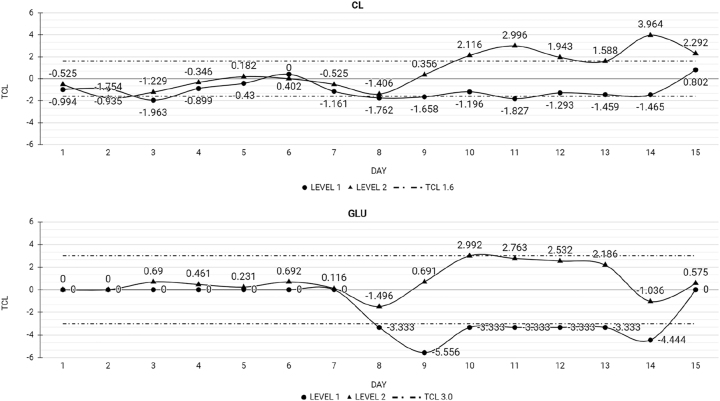
Analytes that lost stability in UC QC in 15 days: CL, chlorine; GLU, glucose; TCL, total change limit; level 1, low-level UC QC; level 2, high-level UC QC.

Tables 2 and 3 show the Xt% for each analyte by day, along with the TCL. The time point at which Xt% exceeded the TCL, thereby resulting in loss of stability, is also provided. A total of 40 of the 48 analytes tested remained stable at the end of the study. In the case of MQ and UC QC, 32 of the 39 analytes remained stable for the 15 study days. In MQ QC, ALP, AST, Ca, LDH, and TBil lost stability at day 8 (ALP), 10 (AST), and 13 (Ca, LDH, and TBil). Figure 1 depicts the behavior of analytes over time. ALP and Ca show a tendency to increase, whereas AST, LDH, and TBil tend to decrease. In relation to UC QC, CL and Glu lost stability at day 8, without showing a clear tendency (Figure 2).

**Table 2:** MQ and UC controls, percentage change for every analyte throughout the study and TCL value.

	Day 1	Day 2	Day 3	Day 4	Day 5	Day 6	Day 7	Day 8	Day 9	D-10	D-11	D-12	D-13	D-14	D-15	TCL
CC MQ																

ALB low	0	−0.92	0	−0.93	0	−2.78	−1.85	−0.93	−0.93	−0.93	0	0	−1.85	−1.85	0.926	**3.43**
ALB high	−1.48	0.74	0	0	0	0	0	0	0	0	0	0	0	−0.74	0	**3.43**
ALP low	0	0.505	1.515	1.515	2.778	3.283	3.283	3.03	**5.051**	3.788	**5.545**	4.293	3.788	3.788	3.788	**4.66**
ALP high	0	−0.50	2.649	3.657	4.035	5.045	4.54	**4.666**	**5.423**	4.54	**4.666**	**4.666**	**4.666**	3.909	4.414	**4.66**
ALT low	1.118	1.616	3.715	0.808	−3.13	1.207	−2.32	0.853	0.519	−1.31	−2	−0.89	−5.48	−4.06	−4.42	**5.64**
ALT high	−0.48	−1.13	−1.45	−2.42	−1.45	−2.26	−2.42	−3.06	−2.90	−2.90	−4.35	−2.90	**−6.46**	−3.71	−5.00	**5.64**
Amylas low	0.002	0.509	0.256	0.002	−0.25	–	−0.51	0.002	−0.25	−0.25	−0.51	0.254	−1.27	0.003	0.254	**3.83**
Amylas high	0.72	1.082	0.841	0.36	0.602	–	−0.72	0.48	0.481	0.481	0.121	0.962	−0.48	−0.60	0.602	**3.83**
AST low	0	−0.39	−1.58	−1.97	−1.58	−1.98	−2.76	−3.95	−5.14	**−7.11**	**−10.7**	**−9.49**	**−13.8**	**−19.4**	**19.76**	**6.12**
AST high	0	0.129	0.124	−0.51	0.383	0.382	−0.13	−2.41	−2.67	−4.57	**−6.73**	−6.10	**−8.38**	**−11.0**	**−11.7**	**6.12**
Ca low	0	−0.324	−0.327	0.647	−0.324	0.003	−1.951	−0.32	−1.631	−0.977	−2.278	−2.278	3.252	3.578	3.252	**4.03**
Ca high	0	0.741	−1.23	1.478	0.494	1.725	0.247	1.724	1.232	0.494	−0.49	−0.00	**8.378**	**9.362**	**8.129**	**4.03**
TChol low	0	−0.17	0.355	0.355	0.176	1.417	1.77	1.063	1.418	1.062	0.531	1.417	−2.12	−1.42	−0.53	**3.41**
TChol high	0	0.122	0.363	0.363	0.726	2.054	2.054	1.087	1.088	1.571	0.967	1.571	−1.69	−0.60	−0.36	**3.41**
CK low	0.619	0.62	−0.25	−0.49	0.124	−1.11	−1.85	−1.47	−0.86	−1.48	−2.59	−1.48	−3.82	−2.34	−1.85	**8.1**
CK high	−1.13	−0.15	−0.71	−1.23	−0.92	−1.54	−2.00	−2.46	−2.67	−2.47	−2.98	−2.62	−4.11	−3.49	−2.93	**8.1**
CL low	−0.33	−0.03	−0.2	−1.40	−0.53	−0.4	−0.07	−0.13	0.067	−1.13	−0.3	−0.3	−1.00	−0.4	−0.03	**2.27**
CL high	1.137	0.287	0.002	0	−0.28	−0.28	−0.28	−0.28	−0.56	0.002	−0.56	−0.57	−0.85	−1.13	−1.13	**2.27**
Crea low	0.172	−0.17	−1.03	−0.86	0.172	–	−1.20	−0.86	−1.72	−1.55	0.696	−0.68	−3.1	−1.20	0.005	**4.4**
Crea high	0.261	0.52	0.983	0.056	–	–	−0.76	−0.40	−0.61	0.312	−0.30	−0.20	−1.39	−1.29	−0.15	**4.4**
cHDL low	0	−2.70	−2.70	−2.70	−0.90	0	0	−2.70	−1.80	−3.60	−5.40	−5.40	−5.40	−5.40	−5.40	**5.89**
cHDL high	0	−1.74	−2.32	−3.49	−0.57	0.585	0.01	−2.32	−2.89	−5.81	−5.81	−5.81	−3.48	−4.64	−4.06	**5.89**
DBIL low	2.381	7.143	7.143	7.143	7.143	7.143	7.143	7.143	0	−2.38	−2.38	−7.14	−7.14	−7.14	−9.52	**19.01**
DBIL high	0	3.846	3.846	3.846	6.41	5.128	1.282	3.846	0	−1.282	0	−1.282	−3.846	−3.846	−3.846	**19.01**
cLDL low	0.273	0.817	−0.54	0	−0.27	0.815	0.546	0.273	−0.27	0.544	0.549	0.817	0.544	0.546	−0.27	**4.98**
cLDL high	2.536	2.929	1.561	1.367	0.002	0.197	0.978	0.585	0.587	0.976	1.369	1.757	2.345	0.975	1.362	**4.98**
GGT low	3.35	2.142	4.194	3.82	2.136	3.408	2.564	2.147	1.72	3.397	3.381	3.82	2.116	0.87	2.537	**6.39**
GGT high	0.769	0.509	1.793	1.308	1.811	1.799	2.32	0.521	1.29	1.29	2.308	2.326	−0.006	3.083	2.829	**6.39**
Glu low	0	0	1.488	1.488	1.786	2.083	1.786	0.893	1.19	1.19	1.786	1.786	−1.786	−0.893	−1.19	**3.09**
Glu high	0	−0.199	1.397	1.994	2.293	2.892	2.592	1.794	1.695	1.595	1.795	2.093	−1.695	−1.196	−0.797	**3.09**
P low	0	0.813	0.813	−0.813	0.813	1.626	0	0	0.813	0.813	1.626	2.439	2.439	2.439	0	**4.72**
P high	−0.457	0.913	0.457	1.37	1.826	1.37	1.37	0.913	0.457	1.37	0.913	0.913	0.913	0.457	0.913	**4.72**
Iron low	0.218	0.218	0.437	0.003	0.436	0.437	0.219	1.525	1.089	1.303	2.391	0.652	−0.22	0.654	0.87	**13.35**
Iron high	1.326	1.62	1.327	1.033	0.593	0.885	0.737	1.177	1.324	1.177	1.031	1.32	0.595	0.737	1.624	**13.35**
K low	0.44	0.177	0.438	0.613	0.525	0.264	−0.60	0.003	−0.34	0.267	−0.08	0.003	−0.52	0.612	0.959	**2.37**
K high	0.228	0.773	0.183	0.545	0.864	0.546	0.091	0.273	−0.50	−0.23	0.272	0.091	−0.54	−0.14	0.228	**2.37**
LDH low	0.56	−0.56	−0.18	−1.12	−3.54	−3.54	−2.80	−2.99	−2.98	−4.10	−3.73	−4.48	**−6.16**	−4.48	**−4.66**	**4.55**
LDH high	1.285	1.045	1.205	0.161	0.241	−0.80	−0.64	0.402	−0.08	−1.12	−0.56	−0.80	−1.05	−0.40	−0.40	**4.55**
Mg low	0	0	0	0	0	0	0	0	−4	0	1.333	0	−4	−1.33	0	**5.71**
Mg high	−1.55	−0.74	0.038	−0.77	−1.55	−1.55	−2.36	0.038	−1.55	−0.74	−0.77	−1.55	−1.55	−0.74	−1.55	**5.71**
Na low	0.243	0.24	0.48	0.48	0.243	0.003	−0.47	−0.23	−0.47	0.247	0.007	−0.23	−0.47	0.48	0.713	**0.92**
Na high	0.214	0.214	0.001	−0.64	0.214	−0.21	−0.42	0	−0.42	−0.42	−0.21	0.001	−0.64	0	0.214	**0.92**
TBil low	0	−1.01	0	−2.02	−3.03	−3.03	−2.02	−3.03	−3.03	−6.06	−9.09	−7.07	**−15.1**	**−15.1**	**−18.2**	**11.27**
TBil high	0	−1.30	−2.17	−2.17	−3.04	−2.17	−3.47	−4.78	−5.65	−6.08	−8.69	−8.69	−9.99	−10.9	**−11.3**	**11.27**
TP low	0	−0.62	−0.62	0	0	0	−1.23	−1.23	−1.85	−1.23	−1.85	0	−0.62	0	1.235	**3.73**
TP high	0.966	0	−0.48	0	0	0	−0.97	−0.48	−1.45	0	0	0	0.483	0.966	0.966	**3.73**
Trig low	0.939	0.941	1.413	1.648	2.118	2.59	2.589	2.354	3.06	4.94	4.237	3.766	4.237	5.411	5.412	**10.37**
Trig high	1.225	1.684	2.725	2.75	3.055	3.212	2.75	2.598	3.981	3.362	3.666	3.667	3.513	3.975	5.04	**10.37**
UA low	−1.12	−0.56	−0.56	−1.11	−0.56	−0.56	−0.56	−0.56	−0.56	−0.56	−0.56	0.565	−0.56	1.13	1.13	**4.55**
UA high	0.721	0.362	1.079	0.362	0.362	0.725	0	0.725	0.362	0.004	0.362	1.083	1.441	1.083	1.8	**4.55**
UN low	−0.79	−0.79	−2.4	−3.21	−3.21	−2.42	0	0.813	0.813	0.019	−0.79	0.019	−0.79	−0.79	−1.59	**7.49**
UN high	0	−1.43	−2.86	−3.33	−3.33	−4.76	0.476	0	0	0	−1.43	0	−1.43	−2.86	−2.86	**7.49**

CC UC																

Ca low	−0.96	0	1.442	0	1.919	1.449	−1.43	0.483	−1.91	2.871	1.925	−0.48	2.374	−2.88	4.79	**15.75**
Ca high	0.002	−0.28	0.572	0	1.714	0.855	−1.14	0.287	0.575	6.572	6.86	6.278	2.289	−2.57	2.574	**15.75**
CL low	−0.99	−0.93	−1.96	−0.90	−0.43	0.402	−1.16	**−1.76**	**−1.66**	−1.20	**−1.83**	−1.29	−1.46	−1.46	0.802	**1.6**
CL high	−0.52	−1.75	−1.23	−0.35	0.182	0	−0.52	−1.41	0.356	**2.116**	**2.996**	**1.943**	1.588	**3.964**	**2.292**	**1.6**
Crea low	1.925	2.129	3.291	0.618	1.784	2.145	0.432	1.539	−1.01	−0.77	0.923	2.256	0.62	0.685	1.686	**12.46**
Crea high	0.954	1.464	2.404	0.686	1.47	2.837	0.04	1.565	−0.02	2.267	4.065	5.399	4.372	−0.272	2.004	**12.46**
Glu low	0	0	0	0	0	0	0	**−3.33**	**−5.56**	**−3.33**	**−3.33**	**−3.33**	**−3.33**	**−4.44**	0	**3**
Glu high	0	0	0.69	0.461	0.231	0.692	0.116	−1.50	0.691	2.992	2.763	2.532	2.186	−1.04	0.575	**3**
IP low	0.749	0.739	3.591	4.439	4.817	4.851	4.32	1.733	0.638	0.749	1.737	4.197	2.607	2.842	3.454	**9.47**
IP high	−0.24	−1.22	0.802	2.202	4.037	3.676	3.246	1.104	1.105	3.616	4.703	6.968	6.482	1.381	1.288	**9.47**
K low	1.021	−2.76	1.531	1.224	1.736	1.534	1.022	0.307	0.205	1.327	−1.53	0.92	0.918	−0.20	−0.41	**12.25**
K high	1.735	1.734	1.831	1.66	3.1	1.213	2.646	2.105	4.553	7.046	9.004	7.649	7.638	7.186	3.149	**12.25**
Mg low	−1.41	−1.17	0.954	0.876	4.212	2.667	−11.5	−2.61	−1.26	−1.70	−4.98	−3.61	−3.46	−1.56	0.799	**20.19**
Mg high	0.519	0.104	1.326	3.325	3.486	4.878	−9.31	0.226	1.972	4.063	4.065	4.904	3.972	−0.53	0.682	**20.19**
Na low	0.155	5.117	1.202	0.581	0.388	0.116	0.698	0.582	−0.35	0.97	4.11	0.931	0.776	0.505	1.435	**14.4**
Na high	0.19	0.379	0.001	0.378	0.754	0.568	0.758	0.564	1.89	4.343	5.661	4.529	4.342	1.135	0.754	**14.4**
UA low	2.836	1.578	3.148	1.572	0.629	0.952	3.774	1.893	0.326	1.261	−0.31	0.003	−0.48	1.258	2.213	**10.48**
UA high	1.852	1.092	2.156	0.777	1.081	2.326	1.7	2.156	1.7	4.156	3.692	4.152	4.002	1.852	1.386	**10.48**
microALB low	0	−1.08	−1.48	0.882	−10.7	−12.1	−10.5	–	−7.15	−1.37	−14.0	−12.8	−1.37	−6.85	4.107	**22.23**
microALB high	0	0.004	−1.14	−0.38	−1.90	−2.29	−3.83	−2.67	−4.98	−1.90	−3.05	−4.58	−20.7	–	−20.7	**22.23**
UN low	2.668	2.322	5.753	2.293	1.576	4.603	−0.87	5.705	4.467	2.637	2.698	3.846	5.645	5.841	4.624	**11.94**
UN high	3.303	2.077	5.391	1.496	1.534	3.311	0.797	5.962	6.762	7.219	8.869	6.978	7.357	1.99	3.941	**11.94**
PT low	−1.65	2.855	2.748	1.981	3.653	−1.65	2.714	−0.55	0.428	2.913	2.339	2.493	2.082	4.251	3.526	**19.6**
PT high	−0.95	0.375	−0.74	−0.10	1.385	0.797	−0.21	0.267	1.647	3.396	3.079	3.604	2.334	−0.24	−1.37	**19.6**
Amylas low	−0.59	0.665	1.923	0.047	0.024	1.923	−0.62	0.665	−1.23	−2.52	−0.62	−1.23	−0.59	0.665	0.665	**47.2**
Amylas high	0.594	0.446	0.741	0.15	0.889	0	0.296	1.187	1.927	3.852	4.594	5.483	4.89	0.445	1.187	**47.2**

TLC, total change limit; “–” result not available due to an error of the analyzer. Bold values, TCL values and values out of TCL.

**Table 3:** LL and LI controls, percentage change for every analyte throughout the study and TCL value.

	Day 1	Day 2	Day 3	Day 4	Day 5	Day 6	Day 7	Day 8	Day 9	Day 10	Day 11	Day 12	Day 13	Day 14	Day 15	Day 16	Day 17	Day 18	Day 19	Day 20	TCL
LL QC																					

Low APO-A	1.8	0.6	0.6	2.1	1.8	1.5	1.5	0.9	1.3	0.6	0.3	0.9	2.5	0.0	1.8	3.7	3.7	3.7	2.5	4.9	**5.5**
High APO-A	–	–	–	–	–	–	–	–	–	–	–	–	–	–	–	–	–	–	–	–	**5.5**
Low APO B	1.9	−2.2	−1.5	−1.9	1.9	0.8	–	−3.4	4.1	6.7	6.0	3.7	4.5	4.0	4.1	3.7	1.1	1.9	2.2	4.1	**7.9**
High APO B	−2.5	−1.0	−1.7	−1.0	1.1	−2.3	–	−2.8	3.9	2.6	2.6	3.7	2.9	3.3	3.5	6.1	4.6	−2.5	5.0	5.4	**7.9**
Low hsCRP	0	−2.1	−4.2	8.3	12	6.2	6.2	6.2	10	8.3	8.3	10	12	25	8.3	4.1	6.2	6.2	10	8.3	**35**
High hsCRP	−2.4	−1.2	−1.8	10.3	9.70	6.67	9.70	6.67	7.27	6.06	8.48	6.06	4.24	6.67	7.88	3.64	5.45	5.45	4.85	7.27	**35**

LI QC																					

Low Hapt	0	−0.9	−1.4	−0.9	−0.5	0.5	0.5	−0.9	2.1	0.9	−0.5	−0.5	−0.5	0.9	0.9	−1.4	−3.8	−2.3	2.8	2.3	**5.8**
High Hapt	0.2	−0.9	−1.1	−1.5	−0.9	−0.2	0.4	−1.8	4.0	3.8	3.1	2	3.8	2.0	3.6	2.9	0.2	−0.6	5.8	**6.2**	**5.8**
Low Trf	−0.2	0	−0.9	0.4	0.4	1.1	1.5	−1.3	−0.6	−0.9	−0.4	−0.2	1.1	−2.6	−2.2	−1.7	−2.2	**−7.8**	**−7.8**	**−8.1**	**5.3**
High Trf	0.8	1.4	1.4	0.0	1.1	2.7	2.9	0.3	0.3	0.4	0.1	0.8	1.9	−1.1	0.8	−0.4	−0.8	−5.2	**−5.9**	**−7.4**	**5.3**
Low CRP	3.6	1.6	2.6	2.1	3.6	1.6	4.7	4.2	2.6	1.6	3.1	−0.5	2.3	0.5	0.5	1.6	0.5	1.6	2.6	4.7	**18**
High CRP	2	3.3	2.6	1.1	2.5	3.7	3.6	2.7	1.9	0.9	1.9	0.3	2.7	1.4	−1.2	1.1	0.1	1.2	1.3	2.3	**18**
Low preALB	−1.0	−0.8	−1.3	−2.9	−3.6	−4.2	2.6	−1.0	3.7	−1.3	−0.5	−1.0	1.3	1.6	−2.0	−1.6	1.3	0.5	−0.8	−2.8	**11**
High preALB	−0.3	0.1	−0.6	0	−2	−0.3	0.1	−0.7	1.5	−1	−1	−0.1	−0.3	0.6	−0.3	−0.6	1.3	0.7	−0.6	−1.8	**11**
Low C4	1.2	1.6	1.2	0.9	0.7	0.7	1.8	1.2	1.3	2.3	0.5	1.8	2.1	0.7	−2.3	−0.2	−1.1	−0.4	−0.5	2.3	**6.7**
High C4	0.4	1.9	1.2	1.3	1.9	3.3	2.3	1.0	2.3	1.4	1.3	1.9	3.2	−1.2	−1.4	0.2	0.1	−1	1.0	0.4	**6.7**
Low C3	−3.2	−0.4	−0.9	−0.9	−0.9	0.5	−1.4	−0.2	−1.3	−0.8	−4.5	−2.7	0.0	−5.4	−5.4	**−7.2**	−5.4	−5	−4.5	−4.0	**5.5**
High C3	−2.4	−0.7	−0.8	−0.8	−1	1.09	−1.4	−1.8	−1.4	−0.6	−2.2	−1.4	0.27	−2.6	−5.2	−2.7	−4.1	−3.2	−2.0	−3.1	**5.5**

TCL, total change limit; “–” result not available, LL level 2 was not estimated for APO-A due to inadequate concentration and because it is not used as internal control of this analyte. Bold values, TCL values and values out of TCL.

**Figure 3:**
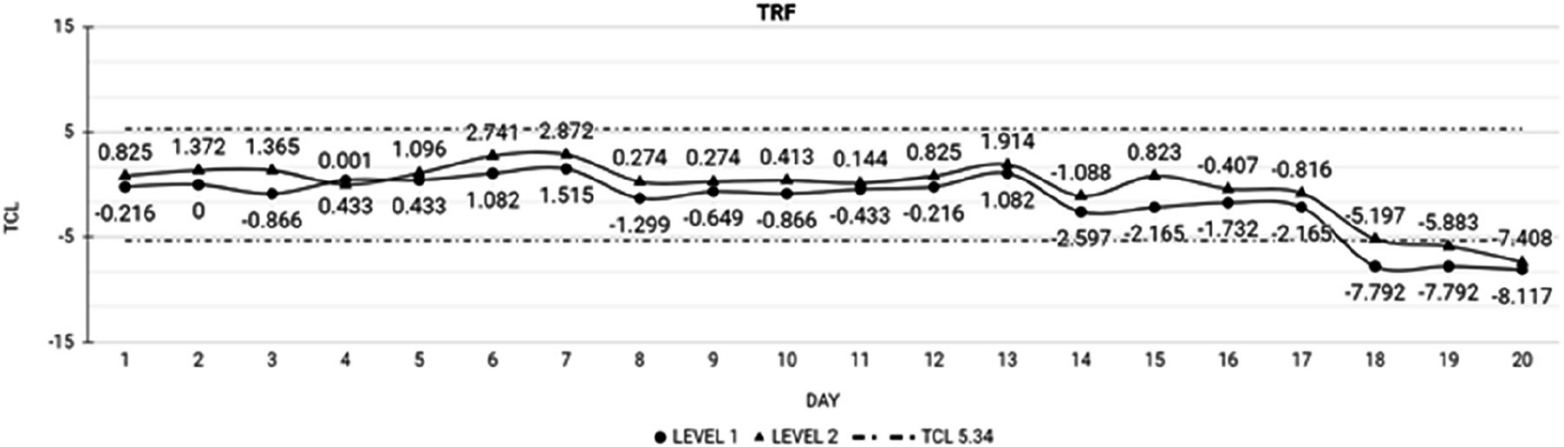
Analytes that lost stability in LI QC in 20 days: TRF, transferrin; TCL, total change limit; level 1: low-level LI QC; level 2: high-level LI QC.

In LI, LL QCs, eight of the nine analytes were stable throughout the 20 days of study, except for Trf in LI, which lost stability at day 18, with a tendency to decrease (Figure 3).

In the case of ALT assayed in MQ and C3 tested in LI, a single Xt% value exceeded the TCL in only a QC level; therefore, it was not considered significant.

## Discussion

This study assessed the stability of a range of biochemical analytes in the QC materials stored in the AS RSM. The 48 analytes remained stable for a minimum of 7 days, based on the stability criterion of TCL. This criterion has been used in similar studies assessing the postanalytical stability of patient’s serum samples stored in automated refrigerated modules and connected to an automated system [3, 4]. In contrast, we tested stability in internal quality control materials stored in the RSM of the analyzer, which influences the analytical phase directly.

To the best of our knowledge, there are only two chemistry analyzers available in the market that incorporate an automated refrigerated module for storage of QC materials that allows automated scheduling. These systems are Siemens AS and Abbott Alinity (Germany). Unlike Alinity, AS has a dedicated RSM for QC and calibrators equipped with magnetic caps. Conversely, in Alinity, QC material and calibrators are stored uncapped in the reagent compartment.

The RSM module saves time to operators and facilitates laboratory work, since QCs can be scheduled to be carried out when the workload is low. Understanding the stability of QC materials stored in the RSM is essential to ensure analytical quality. To date, and to the best of our knowledge, no studies have been published to assess the stability of internal quality control materials stored in the RSM of AS.

Bio-Rad InteliQ control materials were not used in this study because they were not available at the time of the study. However, in our opinion, the results obtained are applicable to current InteliQ controls, since they use the same control materials. Only the container is different, as InteliQ uses 5 mL tube and in the study were used QCs vials that were transferred in 5 mL tubes. The time of stability provided in the instructions for use (IFU) for the QC materials used in this study is consistent with the time indicated in the IFU for InteliQ QC materials. According to Bio-Rad, in MQ QC, all analytes remain stable for 14 days, except for ALP, AST, and TBil, which are stable for 9 days, and DBIL, cHDL, CK, IP, and Trig, which are stable for 7 days. In contrast, the results of this study demonstrate that all analytes remained stable for a minimum of 15 days, except for ALP, which lost stability since day 8, AST since day 10, and Ca, LDH, and TBil since day 13. With respect to UC QC, Bio-Rad IFUs indicate that all analytes are stable for 30 days. In our study, however, UC QCs were assayed for 15 days, and we observed that CL and Glu lost stability since day 8, based on the TLC criterion. Finally, in LI and LL QCs, Bio-Rad IFU indicates that all analytes of the study are stable for 30 and 14 days, respectively. However, the results of this study demonstrate that all analytes remained stable for a minimum of 20 days, except for LI Trf, which lost stability since day 18. These results highlight the necessity to test QC stability based on the criteria established by each laboratory.

In conclusion, the AS refrigerated storage module preserved the QC materials studied appropriately. Awareness of the time of stability of each analyte makes it possible to set the maximum time of use of a QC by analyte and not by control material. Finally, the results of this study enable adequate QC testing by laboratory professionals, thereby reducing the number of repetitions and changes of control material in the presence of biased results.

### Limitations

This study has an analytical limitation due to a possible bias caused by the analysis of control materials in different analytical runs. According to the paper published by Fernández-Calle et al. [7], aliquots should have been used and frozen for analysis in the same run. However, these recommendations were published in 2019, after this study was carried out.
